# Challenges in Transitional Phases of Foster Care for Older Adults: Perspectives of Foster Caregivers

**DOI:** 10.1111/scs.70297

**Published:** 2026-07-17

**Authors:** Sini Eloranta, Teija Juntunen

**Affiliations:** ^1^ Turku University of Applied Sciences Finland; ^2^ University of Turku Department of Nursing Science Finland; ^3^ The Wellbeing Services County of Ostrobothnia Finnish Centre for Client and Patient Safety Finland

**Keywords:** adult foster homes, care transitions, home and community‐based care, residents' safety, thematic analysis

## Abstract

**Aim:**

To examine the challenges associated with transition processes in adult foster care for older adults from the perspective of foster caregivers.

**Design:**

A qualitative design with semi‐structured interviews was used to explore foster caregivers’ experiences of care transitions and the meanings associated with these situations.

**Ethical Considerations:**

Permissions were obtained from a Finnish city, and ethical review was not required according to national guidelines. Participation was voluntary, informed consent was obtained, and confidentiality was ensured.

**Methods:**

Data were collected through five semi‐structured interviews with foster caregivers recruited via the Family Care Association. Interviews were conducted remotely, audio‐recorded, transcribed verbatim, and analysed using inductive thematic analysis.

**Results:**

The findings identified challenges across transition phases. In early stages, some older adults were placed in foster care despite having physical or cognitive impairments exceeding the model's capacity, complicating daily coping. Financial insecurity influenced decision‐making and could contribute to the continuation of care beyond appropriate limits. In later stages, strong attachment relationships developed over time, making the ending of care emotionally demanding and affecting the entire foster care community.

**Limitations:**

The small sample size and single‐country context may limit transferability, and the perspectives of older adults were not included.

**Conclusion:**

Transition processes in adult foster care involve tensions between residents' needs, care model limitations, structural conditions, and emotional demands. Clearer placement practices and stronger support structures are essential to improve care quality and caregiver wellbeing.

## 1 Introduction

Older adult foster care is a care model that integrates both housing and care, and it is used in several countries, including England, France, Italy, the United States, and Belgium [1, 2]. In Finland, the system is administered by the 21 wellbeing services counties, which are responsible for organising social and healthcare services as well as rescue services. Wellbeing services counties are regional public authorities funded by the state. They do not necessarily provide services themselves but ensure their availability, quality, and oversight [3]. In Finland, older adult foster care is provided in accordance with the Act on Social Welfare (1301/2014) [4], while the Act on Family Care (263/2015) [5] defines the legal framework that governs foster care arrangements. This legislation specifies the criteria for foster carers and the maximum number of individuals they may care for simultaneously. In commission‐based foster care, the wellbeing services county enters into a commission agreement with the caregiver. These caregivers are neither employees of the county nor entrepreneurs; instead, the agreement defines the care fee, reimbursement of expenses, and other forms of support they receive [6].

In Finland, the Act on Family Care (263/2015) [5] outlines the fundamental requirements and responsibilities governing foster care, specifying the criteria for foster carers, the maximum number of individuals they may care for at one time, the obligations of the wellbeing services counties in organising foster care, and the mechanisms for overseeing service provision. One foster carer may care for up to four individuals at a time. This number includes preschool‐aged children and other people requiring special care or attention who live in the same household as the carer. Foster caregivers are not required to have social or health care education; they only need to complete a short training course before becoming a family caregiver.

In foster care, older adults live in ordinary homes with foster carers who are not related to them [7, 8]. Foster care is a “semi‐formal” model that blends elements of formal and informal caregiving. Although foster care is guided by official regulations and supported by structured services, the foster carer simultaneously assumes a family‐like role [1].

In Finland, the number of adult foster care homes has increased substantially over the past decade: There were 46 homes in 2012 and 224 in 2022, and during the same period, the number of older adults living in foster care rose from 112 to 2037 [9]. Since 2023, the Resident Assessment Instrument (RAI) has been used to assess the care and support needs of older adults in foster care. These data are not publicly available, but typical challenges faced by older adults in foster care include insecurity, loneliness, early‐stage cognitive impairment, low mood, and difficulties with daily activities such as eating, hygiene, and mobility, all of which may threaten their ability to cope at home. A recent analysis of Finnish care policy documents indicates that foster care is viewed as strengthening the sustainability of the social and healthcare system and providing more cost‐effective forms of care [10].

The growth of adult foster care in Finland raises important questions about the experiences of foster caregivers, underscoring the need for further research.

## 2 Background

Adult foster care is an intermediate form of housing situated between home care and institutional long‐term care, intended for older adults whose needs exceed what home care can provide but who do not require 24‐h supervision [11]. A defining feature is that the family caregiver and the older person live together in the caregiver's private home, forming a small, home‐like care community that differs from institutional settings such as nursing homes [12]. Foster care involves sharing daily life, home, and personal privacy with older adults, and foster caregivers often describe how their private home merges with the public sphere of care [8]. The work is mainly performed by middle‐aged [12] or older women [13]. Because the care takes place in the caregiver's own home, the boundaries between work and leisure often become blurred, particularly in long‐term foster care [1, 14]. Foster care for older people is also often temporary: Care periods may last months or years, but living in a foster home until the end of life is not always feasible, as conditions may deteriorate, requiring transfer to another care setting [15].

Earlier research on older adult foster care is limited [13, 16, 17], but several studies have examined its benefits and challenges. Research has highlighted the well‐being of foster caregivers [8, 16, 18] and the positive outcomes for older residents [12, 16, 17, 19]. Community is often mentioned as a key strength: The home‐like environment supports social relationships, safety, individuality, and everyday participation [19]. Strengths identified in international studies include personal and human‐scale support, caregiver availability and benevolence, high‐quality living environments, nutritious meals, increased participation in daily activities, and the stimulation of autonomy [12]. Close, family‐like relationships often develop, and participation in the family's life may enhance well‐being and reduce feelings of loneliness [16].

Research also identifies considerable challenges. The merging of private and professional spaces may be demanding for caregivers [8]. Both caregivers and residents require time for mutual adaptation [12]. The temporary nature of placements creates emotional strain, including feelings of guilt or inadequacy when care can no longer be provided [14]. Foster caregivers also face financial insecurity when a placement ends, and a new resident is not immediately available [8, 20, 21]. Ending a care relationship is emotionally demanding and may evoke sorrow, guilt, and uncertainty, resembling the experiences of family caregivers [8, 21]. Decisions regarding resident transfers to another care setting require careful timing and sensitivity regarding the needs of residents and families [8].

Although the relevance of adult foster care is increasing, very little research has focused on transition phases, the critical moments when care relationships begin, change, or end. This study addresses this gap by examining foster caregivers' experiences of the transition phase.

## 3 Aim and Objective

The aim of this study is to examine the challenges associated with transition processes in adult foster care from the perspective of foster caregivers. The study aims to generate knowledge that can support the development of care practices, support structures, and service systems within adult foster care. The research question is: What types of challenges do adult foster caregivers encounter during transition processes in foster care involving older adults?

## 4 Methodology and Method

### 4.1 Design

A qualitative design using semi‐structured interviews was chosen as the most appropriate methodology for addressing the research question [22]. The focus of this study was on foster caregivers' subjective and meaningful experiences, particularly those related to care transitions. A semi‐structured interview approach provided a framework while allowing flexibility, enabling participants' voices to be heard. This method supported a deep understanding of the meanings embedded in their experiences. Depth of inquiry, not breadth of generalisability, was the primary goal of this qualitative study [22, 23].

### 4.2 Participant Recruitment

The participants were a convenience sample [24] of five foster carers from a geographically wide area of Finland. The foster carers were recruited via the national Family Care Association. The service manager of the association sent an email to the foster carers on their register, informing them about the study and asking for their willingness to participate. The inclusion criteria were (a) foster carers who had experience as caregivers for older individuals in their own homes, and (b) experience in transition processes. If a caregiver was interested, the service manager provided the researchers with contact details via email. Five foster carers from across Finland were interested in participating in the study. The researcher contacted the participants by telephone and arranged for interviews via Teams.

Participants were asked to provide the following background information: Years of experience as a foster carer, number of long‐term residents, experience with the transition process, and previous work experience in social and health care. Due to the small sample size of five participants, collecting more detailed demographic information was intentionally avoided to protect participant anonymity and privacy.

### 4.3 Data Collection

The data were collected through semi‐structured interviews in autumn 2023. At the beginning of the interview, the researcher once more explained to the interviewee the aims of the study, the voluntary nature of their participation, and the possibility of withdrawing from the study at any time.

The interviews resembled conversations and progressed on the interviewees' terms, while the researcher presented specific questions in accordance with the research aims. The themes were based on the research question but were supplemented with themes related to transition processes identified in the literature [8, 14, 15]. The interview themes focused on transition processes, the challenges associated with these situations, and the foster caregivers' experiences. The researcher encouraged the interviewees to share their experiences as openly as possible, and the interviews were conducted in a confidential atmosphere. During the interview, the researcher was an active listener and judged the need to ask supplementary questions based on the amount of information provided by the interviewee pertaining to the research aims. Questions such as “Could you tell me more about this?” and “Is there anything more you would like to tell me?” were asked. The participants shared their experiences openly and candidly during the interviews. Altogether, the interviews lasted 5 h and 33 min. The interviews were audio‐recorded with the consent of the interviewees. After the interviews, the researcher transcribed the material.

### 4.4 Data Analysis

The data were analysed using qualitative, inductive thematic analysis [25, 26]. First, the data were read multiple times to gain a deep understanding of the interviewees' experiences. The unit of analysis was a sentence or part of a sentence that conveyed the meanings foster carers attributed to their experiences related to transitional situations. These units were coded, and preliminary themes were constructed based on the codes. Each theme was given a name. The preliminary themes were then reviewed and further developed to ensure internal coherence and distinctiveness. The themes were related back to the entire dataset and the research question. The themes reflect key patterns of meaning identified in the data that are relevant to the research question. Finally, the researcher revisited the original data in the last phase of the analysis to ensure that the identified themes genuinely reflected the content of the data.

### 4.5 Rigour

The rigour of the qualitative data analysis was ensured using the criteria proposed by Lincoln and Guba (1985) [27]: Dependability, confirmability, credibility, and transferability. Dependability was ensured through a systematic and transparent analytic process. All interviews were audio‐recorded and transcribed verbatim. The data were analysed using inductive thematic content analysis, progressing step by step from familiarisation with the data to open coding, grouping of codes into subcategories, and abstraction into main categories. Analytic decisions and the development of categories were documented throughout the analysis, enabling the coherence of the analytic process to be assessed.

Confirmability was supported through investigator triangulation. Interpretations of the analysis were continuously discussed among the researchers until a consensus was reached. In addition, confirmability was enhanced through ongoing collaboration and reflexive discussions throughout the analytic process. Credibility was strengthened by the researchers’ professional background and familiarity with the research topic. Transferability was ensured by providing a detailed description of the analytic process; however, participant characteristics were reported only at a general level to protect anonymity.

### 4.6 Ethical Considerations

Appropriate permissions were obtained from a Finnish city. Due to the research design, prior ethical review was not required in Finland [28]. All participants were informed orally that participation was voluntary and that they had the right to withdraw at any time. Orally informed consent was obtained from all participants, and all information was treated confidentially.

## 5 Findings

Older adult foster carers working in their own homes under a commission relationship were interviewed. The participants had worked as foster carers for older people in their own homes for periods ranging from 1.5 to 6 years. All caregivers had at least two long‐term residents and a few places for short‐term residents. Foster caregivers had experiences regarding older people transitioning to enhanced service housing, either from one case or from several over the years. Some foster carers had work experience in the social and health care sectors or in another previous profession.

This section presents foster caregivers' experiences of care relationships with older adults, focusing on challenges at different stages. The findings are organised into two themes: Challenges in the early stages of care, including issues related to the suitability of placements and demanding care situations, and challenges at the end of care relationships, highlighting emotional impacts and the effects on the foster care community.

### 5.1 Challenges in the Early Stages of Foster Care Relationships

#### 5.1.1 Placement of Individuals With Poor Functional and Cognitive Capacity

The functional capacity of older adults moving into foster care was often already too weak for them to survive in the foster home. The residents might need a lot of assistance with daily activities, such as moving from bed to a wheelchair, using the toilet, and eating. This made daily coping in the home environment of the foster home more difficult. Interviewees described situations in the foster home where two assistants, e.g., were needed to move a resident to bed. Interviewees also described that the residents' cognitive capacity was already severely impaired at the time of admission to the foster home.

“They (older adults) are already very forgetful when they arrive and, well, in that sense, they need a lot of help” (2).

Cognitive impairment may have caused some residents to experience restlessness and insomnia. This restlessness negatively affected the entire foster home community. Foster caregivers considered the residents' ability to sleep through the night as the most important criterion for successful foster care. In cases of sleep difficulties, home care could prescribe sleeping medication for the residents. (In foster care, older adults have the right to access other outpatient services according to their care needs.) A source of tension was that the foster caregiver felt the medication was being used to support the success of the foster care arrangement rather than to address the underlying cause of the resident's insomnia. One interviewee mentioned finding trials with strong medication unethical, as they were used to ensure the resident would sleep at night and thus be suitable for foster care.

“They try medication. In a way, I also find it wrong to medicate someone just because that person is in the wrong place” (2).

The interviewees described situations where the older person's relatives had not provided a realistic picture of the person's condition and ability to cope at home prior to the transition to foster care. At the time of data collection, there was no systematic functional capacity assessment system in use during transition situations, such as RAI (Resident Assessment Instrument), which the interviewees believed would support the evaluation of older adults' functional capacity and service needs by providing a more accurate and realistic understanding of the residents' care needs.

“I really hope RAI will be introduced in foster care soon” (5).

Overall, challenges in the early stages were associated with situations in which some older adults arrived with such severe physical or cognitive impairments that foster caregivers felt they would be unable to manage everyday life in the family home.

#### 5.1.2 Adaptation to Challenging Care Situations

The adaptation to challenging care situations was partly influenced by foster caregivers' fear of income loss. If there was no certainty of a new resident replacing the one moving out, the risk of losing income could result in the frail older person not necessarily being transferred, but continued providing still being cared for in foster care.

“All of a sudden, you might have three people moving out, and that's a financial disaster” (5).

Ensuring the livelihood of the foster caregiver was something the interviewees described with some apology, but in their experience, this is a reality that must be considered.

To secure their livelihood, foster caregivers were more willing to accept residents with lower functional or cognitive capacity into foster care. The foster caregiver sometimes had to weigh their own financial situation against the best care option for the resident. This could lead to caregiver burnout.

“When applying for a place in enhanced service housing, we have been quite exhausted every single time” (4).

Foster caregivers had a strong desire to manage their caregiving responsibilities independently and were often reluctant to accept outside help in their home. Even when a resident was no longer considered suitable for foster care, caregivers reported having become accustomed to the situation and continuing to provide care to the best of their ability.

“Substitute caregivers (assigned during statutory leave) have already said that they don't think the resident belongs in foster care anymore” (5).

The interviewees wondered how best to find residents suitable for foster care. Their experience was that people live at home for too long, and their functional capacity is no longer sufficient to manage daily life in foster care. When an older person's functional or cognitive capacity was too low for the daily life of the foster home, it threatened the physical and mental endurance of the foster caregiver.

“It was difficult. At night, he would go to the toilet and had already done it there. In the end, it got messy, also on the bed” (1).

If an older person's functional or cognitive capacity was too low for the daily life of the foster home, this led to a short stay in foster care, and the resident had to move to a nursing home. Thus, the residents of a foster home often changed. It was felt that in transfer situations, a reliable assessment of the resident's functional ability and service needs, conducted using a validated evaluation tool such as RAI, might support the suitability assessment for family care.

As these findings demonstrate, although the care needs of the residents at times exceeded the caregiving capacity of the family care home, the foster care caregivers adapted to the situation. They described continuing the care relationship because they had a strong desire to manage their caregiving responsibilities, or due to financial dependence.

### 5.2 Challenges in the End‐of‐Care Relationship

#### 5.2.1 The Moment of Ending the Care Relationship

The ending of the care relationship was perceived as challenging because of the strong attachment developed with the resident. The interviewees described forming close bonds with the residents. Ending the care relationship with an older person who had lived in foster care for years was particularly difficult.

“In foster care, you are so deeply immersed in the daily life. Letting go is terribly difficult” (1).

Some interviewees felt that they wanted to maintain a certain distance from the foster care residents to make it easier to let go when the time came.

The interviewees described not only challenges with going through the process of ending the care relationship with the older person moving out, but also with having to let go of the resident's relatives, who might have become very important to the foster caregiver.

“When you let go of that person, you don't just let go of them. You also let go of their close ones who have often become friends” (3).

In the interviews, all caregivers mentioned that they were happy to hear about their former residents. Since most of the residents who moved out had cognitive impairment, communication with the residents often took place through their relatives and close ones.

Overall, the findings suggest that long‐term care relationships often led to the development of important and meaningful bonds between the foster caregiver, the resident, and the resident's relatives. These long‐standing relationships made the process of letting go emotionally challenging.

#### 5.2.2 Impact on the Foster Care Community

The departure of a resident from the foster home also affected other residents. Other residents might miss the resident who moved out.

“Even a person with cognitive impairment can remember that they have experienced that kind of friendship, and it feels really bad that we have to break those friendships. That we can no longer care for them here. You also have to go through those feelings and support them as they grieve” (4).

The community went through the process of letting go together, discussing and adapting to the new situation. Foster caregivers found ending the relationship of a resident demanding and felt their own endurance was being tested.

“It was difficult. It felt like a family member was leaving. It's not the easiest thing to say that they have to leave” (1).

The interviews revealed that the foster caregiver might feel a sense of failure for not being able to care for the resident who moved out. When residents moved out, the foster caregivers described a new daily life beginning in the foster care home, possibly with the arrival of a new resident. The process of ending the care relationship of the resident moving out was still ongoing, and a new resident had to be welcomed.

“Even a person with a memory disorder may remember that they have been able to experience that kind of friendship, and it feels very painful that we have to break those friendships. That we are no longer able to care for them in our home. One also has to go through such emotions oneself and provide support while they are doing that grief work” (H4).

In this sub‐section, the findings reveal that foster caregivers felt that the ending of a care relationship affected the entire family‐like community and challenged their own endurance. Letting go also evoked feelings of failure.

## 6 Discussion

This study examined the challenges of transition processes in adult foster care from the perspective of foster caregivers. Adult foster care has received limited research attention, and prior studies have rarely focused on transition processes and caregivers' experiences of them. By foregrounding foster caregivers' perspectives, this study addresses an important gap in the literature and demonstrates that transitions are complex processes with significant ethical, emotional, and practical implications.

### 6.1 The Admission of Individuals With Increasingly Complex Care Needs Into Adult Foster Care

Our findings reveal that some residents entered adult foster care in such poor physical or cognitive condition that they were no longer able to manage everyday life. This finding is consistent with recent research showing that the profile of adult foster care residents increasingly resembles that of long‐term institutional care, particularly regarding dementia and high levels of care dependency [13]. Institutional long‐term care is typically characterised by continuous care provision, professional staffing, and access to clinical and organisational support structures required to meet complex and intensive care needs [17]. In contrast, adult foster care has traditionally been intended for older adults who do not yet require round‐the‐clock care [11]. When residents' functional capacity falls below this threshold, the adult foster care model may no longer be able to adequately meet individuals' needs.

Within ageing policy, adult foster care is positioned as part of ageing‐in‐place strategies and home‐like care arrangements that aim to provide a non‐institutional alternative to long‐term residential care [3]. However, the findings of this study, in line with previous research [13, 16], suggest that adult foster care practices are drifting away from this original ideal when individuals with care needs comparable to those in institutional care are placed in foster homes. This shift raises both ethical and practical concerns, as foster caregivers may become responsible for care tasks that exceed their skills, resources, and capacity within a non‐professional caregiving role. In this respect, our findings echo earlier observations that the boundary between adult foster care and institutional long‐term care has become increasingly blurred [1, 8].

The findings raise questions about whether adult foster care is increasingly functioning as a substitute for institutional care without the corresponding professional staffing, clinical oversight, and structural support that characterise institutional settings. Previous studies have shown that such arrangements may place foster caregivers under increasing pressure, exposing them to heightened care burden and responsibility while operating outside formal professional frameworks [1, 18]. This development may have adverse consequences for both caregiver wellbeing and resident safety. Overall, our findings point to a gradual erosion of the distinction between adult foster care and long‐term institutional care, suggesting a shift that challenges the original positioning of adult foster care as a non‐institutional, home‐like form of support for older people.

### 6.2 Suitability of Placements in Adult Foster Care

In this study, interviewees reflected on the challenge of identifying suitable foster care residents to ensure successful placements. Foster care is associated with the assumption of ordinary family life and does not require professional basic training [1, 14]. At the same time, foster caregivers are responsible for the wellbeing of their residents around the clock, and the task is binding [18]. Foster care is therefore not appropriate for everyone, and there may be situations where an older adult requires 24‐h care or nursing [20].

The findings demonstrate that the identification of a suitable resident is a pivotal factor in adult foster care. Inappropriate placements may jeopardise the smooth functioning of everyday life in the foster home and negatively affect the wellbeing of both residents and caregivers. These findings highlight tensions inherent in adult foster care, which is positioned between ordinary family life and increasingly complex care needs, and underscore the challenges associated with assessing suitability within this care model [1, 11].

### 6.3 The Continuation of Foster Care Beyond What Was Appropriate

In this study, foster caregivers expressed concern about potential income loss in situations where a departing resident was not expected to be replaced. Similar observations have been reported in previous studies [8, 18]. In Finland, compensation for foster caregivers is directly determined by both the number of residents and the intensity of care required [7], which makes caregivers' livelihoods highly dependent on the continuity of care placements.

In some cases, this situation led foster caregivers to continue care relationships with individuals who were no longer suitable for foster care, potentially creating risks to residents' safety and compromising caregivers' wellbeing [8, 18]. Foster caregivers often found themselves balancing financial security, responsibility, and the quality of care [1]. Importantly, this tension should not be viewed solely as a matter of individual decision‐making, but rather as reflecting a structural incentive trap in which discontinuing an inappropriate care arrangement may result in immediate income loss, thereby hindering necessary transitions. In such situations, caregivers may be placed in a position where economic security and the provision of appropriate and safe care are in direct conflict.

The Finnish compensation system, based on the number of residents and the intensity of care required [7], may reinforce this mechanism and unintentionally incentivise the continuation of care arrangements beyond their appropriate limits. Overall, the findings suggest that structural and financial factors may sustain care placements that no longer meet residents' needs, raising concerns about the sustainability, equity, and safety of adult foster care within the broader care system for older adults [1, 8].

### 6.4 The End of Care Relationships and Moral and Emotional Implications

The ending of a care relationship evoked feelings of failure and guilt among foster caregivers, and caregivers clearly expressed a need for support. From a care ethics perspective, caregiving involves a moral commitment and an ongoing responsibility toward another person, grounded in attentiveness, responsibility, and relational bonds [29]. Within this framework, ending a care relationship may be experienced as a moral rupture rather than as a purely practical transition, which helps to explain why feelings of guilt may arise even when the decision is justified in terms of the resident's safety.

Previous research has similarly shown that the termination of care relationships can be emotionally burdensome for foster caregivers and may generate sorrow, guilt, and uncertainty, particularly in long‐term and close caregiving relationships [1, 8, 21]. Such experiences can also be understood in relation to moral distress, which occurs when caregivers recognise the ethically appropriate course of action but feel constrained by structural, organisational, or economic factors [30]. In the context of adult foster care, moral distress and emotional burden appear to accumulate particularly during transition points, where decisions about continuing or ending care involve ethically complex choices in the absence of adequate structural and professional support.

Our findings extend previous research by highlighting that the support needs expressed by foster caregivers should not be interpreted as signs of individual vulnerability or insufficient coping. Rather, they reflect structural characteristics of a care model in which emotional labour and moral responsibility are central but often remain unrecognised and insufficiently supported [1, 18]. This underscores the importance of considering emotional and ethical dimensions as integral features of adult foster care, particularly during transition processes.

## 7 Strengths and Limitations

The strength of this study lies in its perspective on care transitions in adult foster care, focusing specifically on critical transition phases that have received limited attention in previous research. By foregrounding the experiential expertise of foster caregivers, the study captures nuanced, practice‐based insights derived from their direct and sustained involvement in caregiving across transition points. Furthermore, by addressing an under‐researched phenomenon, the study contributes novel insights into the literature on adult foster care and advances understanding of transition processes within this care setting.

However, several limitations should be acknowledged. The sample size was small, and participant diversity was limited, which will have limited the breadth of experiences captured. Furthermore, the study was conducted in one country within a specific legislative context, which may limit the transferability of the findings to other settings. In addition, the perspectives of older people themselves were not included, representing an important gap in understanding care transitions in adult foster care.

These limitations indicate directions for future research, highlighting the need for further qualitative studies involving a larger and more diverse group of foster carers, as well as the inclusion of older adults' perspectives, to deepen understanding of transition processes in adult foster care.

## 8 Conclusion

Overall, the findings suggest that transition processes in adult foster care involve persistent tensions between residents' care needs and the intended care model, financial and structural conditions, and the emotional demands associated with ending care relationships. These challenges are interconnected rather than isolated and reflect broader structural features of adult foster care. Together, the findings highlight the importance of clearer placement practices and more consistent support structures that recognise the ethical, emotional, and relational dimensions of foster caregiving to enhance the sustainability, safety, and fairness of adult foster care within ageing care systems.

## 9 Implications for Clinical Practice, Policy, and Future Research

As the global population ages rapidly, the need to identify sustainable alternatives to traditional long‐term care is becoming increasingly urgent. Adult foster care may represent a promising and more home‐like option alongside conventional nursing homes and institutional care facilities.

Challenges related to inappropriate placements, prolonged care beyond suitability, and the moral and emotional burden associated with transitions underscore the need for clearer and more coherent frameworks guiding adult foster care practices. The findings point to the importance of nationally consistent eligibility and suitability criteria and systematic assessments prior to placement to ensure better alignment between residents' needs and the foster care model. In addition, more unified national practices and support structures may strengthen the operational conditions of adult foster care and reduce regional variation in care trajectories. The findings reveal that existing financial structures may unintentionally shape care decisions and create pressures to continue care beyond what is appropriate. Addressing these structural conditions is essential to support foster caregivers' wellbeing and to ensure that care decisions prioritise resident safety and care quality.

Future research should further explore how eligibility criteria, assessment practices, and funding models interact with caregivers' experiences, wellbeing, and ethical decision‐making. Longitudinal and comparative studies could provide valuable insights into how adult foster care can be better aligned with both policy ideals and the complex realities of care transitions in ageing societies.

## Author Contributions

Study design: S.E.; T.J.; Data Collection: T.J.; Data Analysis: S.E.; T.J.; Manuscript Preparation: S.E.; T.J.

## Funding

The authors have nothing to report.

## Ethics Statement

Ethical approvals were sought and obtained from a Finnish city.

## Conflicts of Interest

The authors declare no conflicts of interest.

## Data Availability

Research data are not shared.
